# Camptocormia as a Novel Phenotype in a Heterozygous *POLG2* Mutation

**DOI:** 10.3390/diagnostics10020068

**Published:** 2020-01-26

**Authors:** Diana Lehmann Urban, Leila Motlagh Scholle, Kerstin Alt, Albert C. Ludolph, Angela Rosenbohm

**Affiliations:** 1 Department of Neurology, Ulm University, 89081 Ulm, Germany; 2 Department of Neurology, University of Halle/S., 06120 Halle, Germany; 3 Genetikum, 89231 Neu-Ulm, Germany

**Keywords:** mitochondrial myopathy, polymerase gamma 2 (POLG2), camptocormia, mutations of nuclear origin

## Abstract

Mitochondrial dysfunction is known to play a key role in the pathophysiological pathway of neurodegenerative disorders. Nuclear-encoded proteins are involved in mtDNA replication, including DNA polymerase gamma, which is the only known replicative mtDNA polymerase, encoded by nuclear genes Polymerase gamma 1 (*POLG*) and Polymerase gamma 2 (*POLG2*). *POLG* mutations are well-known as a frequent cause of mitochondrial myopathies of nuclear origin. However, only rare descriptions of *POLG2* mutations leading to mitochondriopathies exist. Here we describe a 68-year-old woman presenting with a 20-year history of camptocormia, mild proximal weakness, and moderate CK increase. Muscle histology showed COX-negative fibres. Genetic analysis by next generation sequencing revealed an already reported heterozygous c.1192-8_1207dup24 mutation in the *POLG2* gene. This is the first report on a *POLG2* mutation leading to camptocormia as the main clinical phenotype, extending the phenotypic spectrum of *POLG2* associated diseases. This underlines the broad phenotypic spectrum found in mitochondrial diseases, especially in mitochondrial disorders of nuclear origin.

## 1. Introduction

Mitochondrial diseases are associated with a wide spectrum of different clinical phenotypes, ranging from mild to severe [1]. They can either be caused by mutations in the mitochondrial DNA (mtDNA) itself or due to mutations of nuclear origin: (I) nuclear genes encoding for enzymes involved in mitochondrial nucleotide synthesis (*TK2, SUCLA2, SUCLG1, RRM2B, DGUOK*, and *TYMP*) or (II) those required for mtDNA replication (*POLG* and *TWNK*) [2]. mtDNA replication is accomplished by DNA polymerase gamma, a heterotrimer consisting of one catalytic subunit of DNA polymerase encoded by *POLG* (alternative gene symbol: *POLG1*) and a dimer of accessory subunits encoded by *POLG2*, a processivity factor for the DNA polymerase [3,4,5]. *POLG* mutations are well-known to cause either autosomal dominant (ad) or recessive (ar) mutations associated with a broad phenotypic spectrum and may account for up to 25% of all adult presentations of mitochondrial diseases [6]. Since the first publication of *POLG* mutations leading to mitochondrial myopathies [7], over hundred different *POLG* mutations have been identified with a wide range of phenotypical presentations. However, only rare descriptions of *POLG2* mutations leading to mitochondriopathies exist [5,8,9,10,11]. Camptocormia (paravertebral muscle weakness with disabling flexion of the spine in upright position) has been described in case reports and case series in association with Parkinsonian disorders, dystonia, and infrequently in functional neurologic disorders [12]. Only in a few cases, camptocormia has been reported in association with myopathic and/or mitochondrial defects [13,14,15,16,17,18,19]. Here, we present a novel phenotype in an already reported heterozygous *POLG2* mutation [2] with the clinical leading symptom of camptocormia, extending the phenotypic spectrum of *POLG2* related mitochondrial myopathies.

## 2. Materials and Methods

### 2.1. Clinical Description

**Figure 1:**
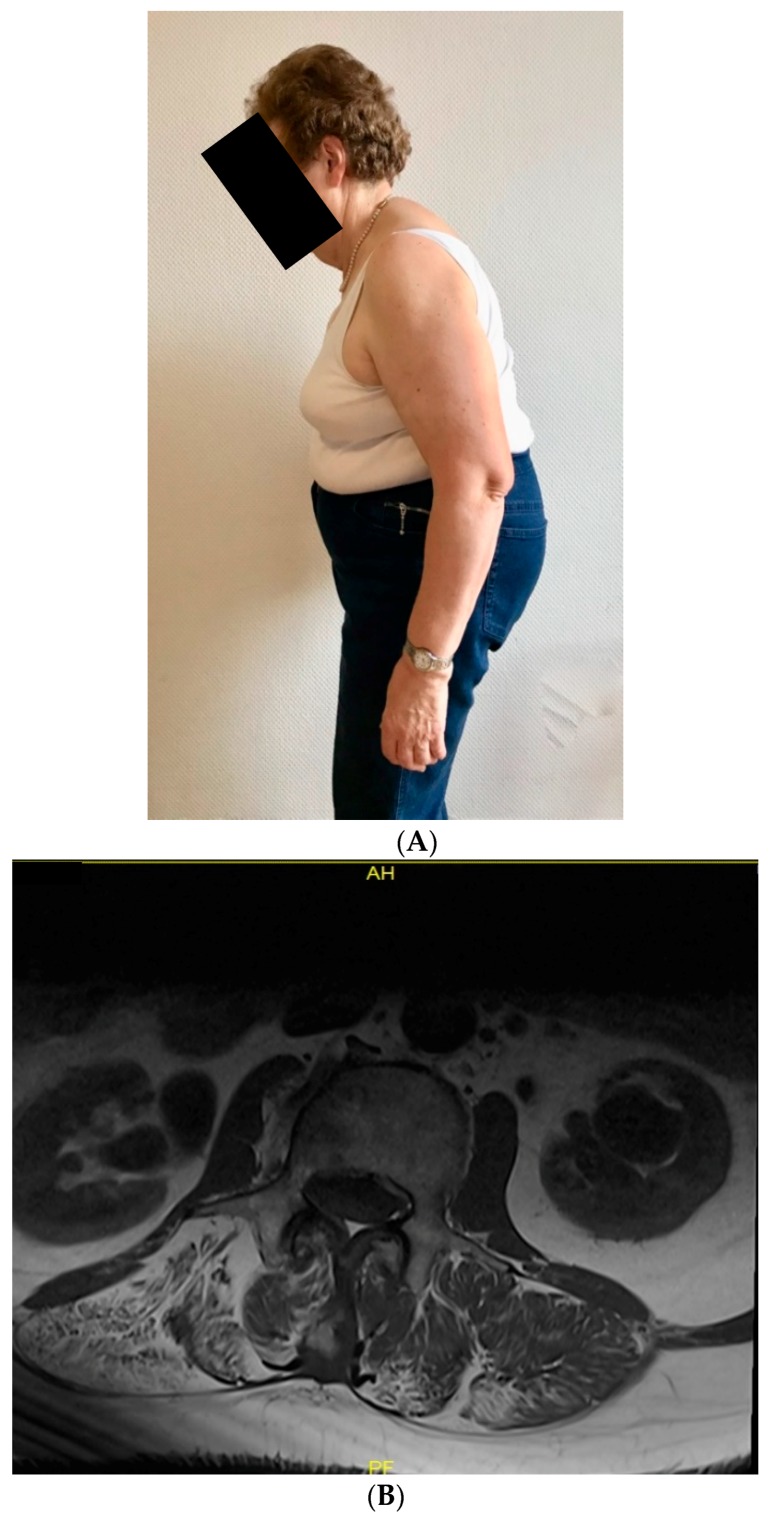
(**A**) Camptocormia in the patient as the main clinical symptom: upright standing is only possible with bended knees, indicating the severity of camptocormia and (**B**) transversal T1- weighted spinal MRI showing a significant fatty degeneration of the paravertebral lumbar spine muscles.

A 68-year-old caucasian female presented with a lumbar kinking camptocormia (Figure 1A), developing over the last 20 years. Walking was only possible using the help of walking sticks, self-erecting was only possible for a few seconds. Laboratory testing showed a mild CK increase (308 U/L , controls < 180 U/L ). Her medical history included chronic pain syndrome, left convex lumbar scoliosis, arterial hypertension, hypercholesterolemia, polyneuropathy and arthrosis of the small finger joints. Clinical examination revealed proximal weakness of the upper limbs (MRC 4/5) and pronounced weakness of the paravertebral lumbar spine muscles with a lumbal camptocormia of 30–40° deviation from the vertical orientation. No ptosis or ophthalmoplegia were found. Deep tendon reflexes were normal and symmetrical. No Babinski sign. Spinal MRI showed a significant fatty degeneration of the paravertebral lumbar spine muscles, no evidence of muscle edema or pathologic CM-uptake indicating florid disease activity (Figure 1B). Electromyography showed chronic neurogenic changes. The patient has three healthy children, with no history of muscle disease, however a neurological examination has been declined. The parents of the index- patient died at higher age without evidence for neuromuscular disorder.

### 2.2. Muscle Histopathology

Cyrostat sections were cut from transversely orientated muscle blocks from the lumbal erector spinae muscle and subjected to standard histological and histochemical analysis including COX, succinate dehydrogenase (SDH), and COX-SDH oxidative enzyme reactions.

### 2.3. Activities of Respiratory Chain Complexes

Activities of respiratory chain complexes were determined spectrophotometrically according to standard protocols [20].

### 2.4. Next Generation Sequencing

To analyze the coding areas as well as 25 bp of flanking intronic sequence of 252 genes associated with different types of myopathies including genes for nuclear encoded mitochondriopathies, genomic DNA was extracted from a patient’s EDTA-blood sample with the FlexiGene DNA Kit following the manufacturer’s protocol (http://www.qiagen.com/). Subsequently, target enrichment was performed with the HaloPlex Target Enrichment System (Protocol Version D, Agilent Technologies, Santa Clara, CA, USA) with an input of 50 ng DNA. After library pooling, next generation sequencing was performed on the Illumina NextSeq 500 System (Illumina, San Diego, CA, USA) with 150 base pair (bp) paired-end reads. The *megSAP* pipeline was used for bioinformatical analysis [21]. Adapter sequences were removed with *SeqPurge* [22], trimmed reads were aligned with *BWA-MEM* [23] to the human reference genome hg19/GRCh37 and improved by indel realignment with *ABRA* [24], yielding an average coverage of 700 reads. Variants were called with *freebayes* [25]. All regions that lacked sufficient read depth (<5 reads) were excluded from analysis. All variants with a percentage share of less than 10% were initially not considered for further analysis. Variant annotation was performed by *SnpEff* [26]. To obtain allelic frequencies, all variants were annotated with several public databases such as ExAC [27] and 1000 genomes [28] as well as our in-house database and databases for clinical significance like *ClinVar* [29]. For the prioritization of potentially clinically relevant variants, *GSvar* (part of ngs-bits [30]) was used to remove all variants with allelic frequencies higher than 1% in any annotated public database or common occurrence in our in-house database (>20) unless they were labeled as pathogenic. Nomenclature of the Human Genome Variation Society (HGVS) was applied to describe the identified variants [31].

## 3. Results

### 3.1. Clinical Findings and Muscle Biopsy

The combination of clinical examination, showing proximal weakness of the upper limbs (MRC 4/5), together with camptocormia (Figure 1A,B) and mild CK increase (308 U/L , controls < 180 U/L ) lead to the differential diagnosis of mitochondrial and other metabolic disorders.

**Figure 2:**
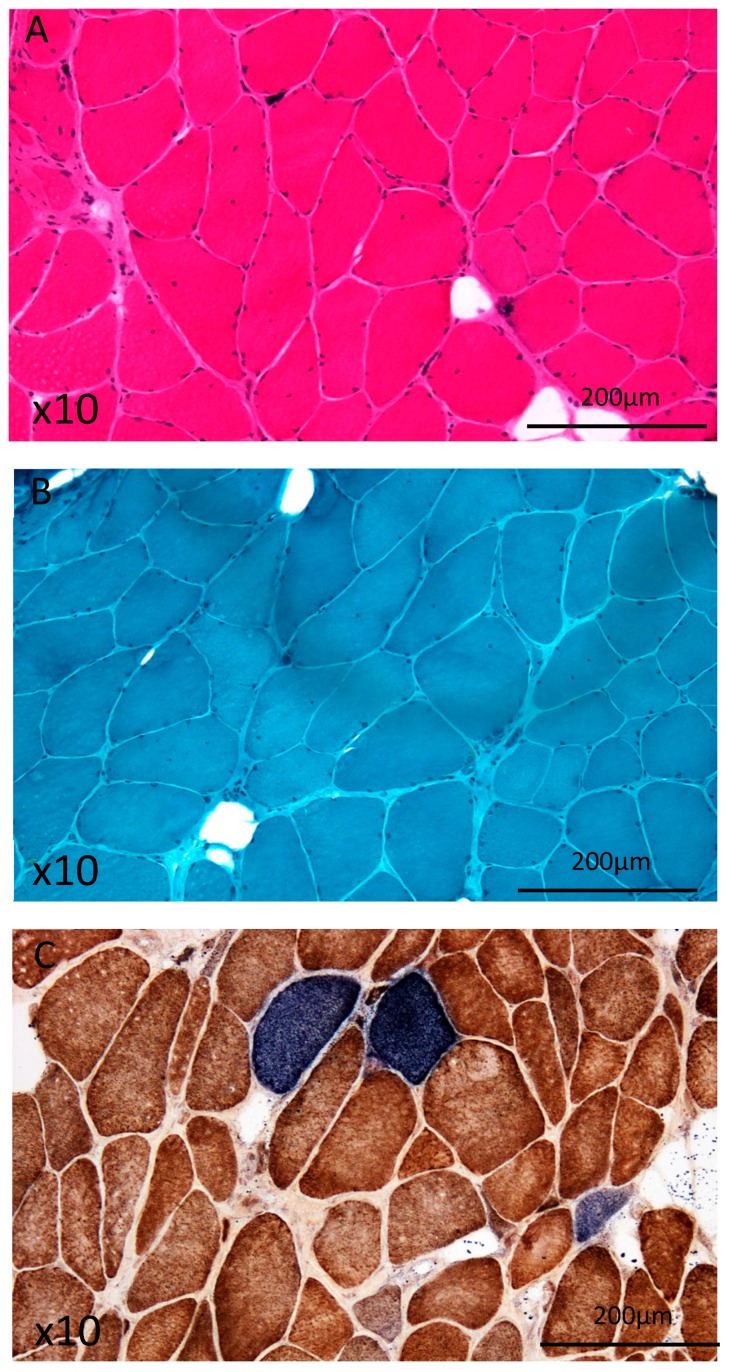
Histochemical findings: (**A**) Hematoxylin and eosin staining, (**B**) Gomori trichrome staining and (**C**) cytochrome c oxidase (COX)-SDH histochemistry revealing numerous COX-deficient (blue reaction product) fibres.

A lumbal erector spinae muscle biopsy showed myopathic changes, including central nuclei, endomysial fibrosis, elevated fiber caliber variation and COX-negative muscle fibers, so as occasional cores. Interestingly, no evidence for ragged red fibres (RRF) was found (Figure 2). Due to the findings of COX-negative fibers and cores, genetic testing has been initiated.

### 3.2. Activities of Respiratory Chain Complexes

Measurements of respiratory chain activities showed decreased activities of respiratory chain complexes I, II + III, and IV (Table 1). Furthermore, citrate synthase was noticeably decreased in patient’s muscle, too.

### 3.3. Genetic Analysis

Due to central cores in oxidative staining, gene panel analysis using next generation sequencing (NGS) was initiated without prior mtDNA sequencing. NGS was performed according to standard protocols and showed no pathological mutations in 251 myopathy-related genes including RYR1, POLG and other nuclear encoded mitochondrial genes (supplementary materials). Hence, the possibility that the camptocormia resulted from mutations in any of these 251 genes was ruled out. Analysis of *POLG2* gene revealed an already reported heterozygous c.1192-8_1207dup24 mutation [8]. Unfortunately, the analysis of further patient material or from family members has been declined.

## 4. Discussion

Mitochondrial diseases are known to show a broad phenotypic spectrum, like varying age-of-onset range. The first reported *POLG2* case was documented in a patient with adult-onset autosomal dominant (ad) progressive external ophthalmoplegia (PEO) as well as COX-deficient muscle fibres and multiple deletions in the mtDNA [9]. Later on, three pediatric- onset autosomal dominant disease cases with varying phenotypes (one young adult with adPEO in late teens and two unrelated infants developing hypotonia, seizures and liver disease) were reported [11]. An infant with fulminant hepatic failure and mitochondrial DNA depletion caused by a homozygous *POLG2* missense variant has been reported [5]. A Belgian pedigree presented with adult-onset cerebellar ataxia, axonal peripheral ataxic neuropathy and tremor in variable combination with parkinsonism, seizures, cognitive decline and opthalmoplegia [10]. The first patient reported with the heterozygous c.1192-8_1207dup24 mutation clinically presented with late-onset ptosis and myopathy [8]. These findings clearly underline the wide phenotypic spectrum, especially in mitochondrial diseases due to nuclear origin, e.g., *POLG* mutations.

Here, we report a novel phenotype caused by an already known *POLG2* mutation. Camptocormia has already been, however only rarely reported in association with myopathic and mitochondrial defects [13,14,15,16,17,18,19]. Until now, camptocormia has not been described in the phenotypic spectrum of mitochondrial myopathies caused by *POLG2* mutations.

Gomori trichrome staining has been postulated as the most sensible and reliable method for detection of mitochondrial abnormalities [32]. Recently, 19 patients harboring *POLG* mutations were studied [33]. Histochemical abnormalities were found in 17/19 cases: cox-negative fibers in 13 cases (68.4%) and ragged red fibers in 12 cases (63.2%) [33]. Interestingly, in the presented case, muscle histology showed no evidence for ragged-red fibres (RRF). This is consistent with a recent study, in which 11 patients with multiple deletions due to *POLG* mutations were analyzed according to frequency of histopathological changes [34] and only 2% of fibres from patients with *POLG* mutations were classified as RRF. However, in this study it has not been clarified which muscle has been taken for biopsy.

The only slight evidence for mitochondrial dysfunction shown in histopathology in the presented case is consistent with the mild clinical phenotype, which coincides with the recently reported patient with the same mutation [8]. The reported mutation was found to cause missplicing and loss of exon 7 in myoblast cDNA due to the 24 bp insertion into exon 7 [8]. However, in none of the reported *POLG2* mutations, camptocormia has been described. Recently, limb muscle biopsy has been recommended as a diagnostic procedure in camptocormia [35]. However, in the presented case, a biopsy of the lumbal erector spinae muscle was performed.

The evidence of cores in muscle histopathology is usually indicational for central core disease (CCD), a congenital myopathy caused by mutations in the gene encoding ryanodine receptor type-1 (RYR1) with muscle weakness defined pathologically by the presence of extensive areas in muscle fibres that are devoid of oxidative enzyme activity (“central cores”) [36,37,38]. Up to date, evidence of cores in patients with mitochondrial diseases due to either mutations of the mtDNA or due to nuclear origin have not been reported. Interestingly, muscle histopathology of the patient presented in this study showed evidence of occasional cores.

Our finding substantially adds to the spectrum of differential diagnostic considerations in patients with camptocormia and histopathological evidence of cores. This strongly underlines the importance of skeletal muscle biopsy as a major diagnostic tool in patients with suspected mitochondrial disease.

## Figures and Tables

**Table 1:** Enzyme activity of respiratory chain complexes, showing decreased activities of respiratory chain complexes I, II + III and IV, as well as decreased citrate synthase activity in the patient muscle.

Respiratory Chain Complexes	Enzyme Activity (U/g Tissue)	
	Patient	Controls (*n* = 20) Mean ± SD (Range)
Complex I	0.125	0.9 *±* 0.6 (0.35–2.5)
Complexes II + III	0.48	1.8 *±* 0.8 (0.8–2.6)
Complex IV (COX)	1.8	10.3 *±* 1.5 (8.2–12.4)
Citrate synthase	0.34	8.4 *±* 2.7 (4.0–11.2)

## Supplementary Materials

Supplementary materials can be found at https://www.mdpi.com/2075-4418/10/2/68/s1.
