# Supplementary material for: Camptocormia as a Novel Phenotype in a Heterozygous POLG2 Mutation
Source: Diagnostics (Basel). 2020 Jan 26;10(2):68. doi: 10.3390/diagnostics10020068 (PMC7168901; doi:10.3390/diagnostics10020068)
Supplement: Supplementary file 1 [file diagnostics-10-00068-s001.pdf]

Genes analysed using nextgenerationsequencing (appearance in alphabetical order):

ABHD5, ACADM, ACADS, ACADVL, ACTA1, ACTC1, ACTN2, AGK, AGL, AGRN, ALDOA, ALG13, ALG14, ALG2, AMPD1, ANK2, ANKRD1, ANO5, APTX, ASAH1, ASCC1, ATP2A1, ATP7A, B3GALNT2, B4GAT1, BAG3, BICD2, BIN1, BSCL2, C10orf2, C12ORF65, CACNA1A, CACNA1S, CACNB4, CAPN3, CAV3, CCDC78, CFL2, CHAT, CHCHD10, CHKB, CHRNA1, CHRNB1, CHRND, CHRNE, CHRNG, CHST14, CLCN1, CNTN1, COL12A1, COL13A1, COL4A1, COL4A2, COL5A1, COL5A2, COL6A1, COL6A2, COL6A3, COLQ, COQ8A, COX15, CPT2, CRYAB, CSRP3, DAG1, DES, DGUOK, DMD, DNA2, DNAJB6, DNM2, DOK7, DOLK, DPAGT1, DPM1, DPM2, DPM3, DYSF, EMD, ENO3, ETFA, ETFB, ETFDH, FAM111B, FBP1, FHL1, FKBP14, FKRP, FKTN, FLNC, G6PC, GAA, GBA2, GBE1, GFPT1, GLA, GMPPB, GNE, GYG1, GYS1, HADH, HADHA, HADHB, HINT1, HNRNPDL, HSPG2, IGHMBP2, ISCU, ISPD, ITGA7, JPH2, KBTBD13, KCNA1, KCNE1, KCNE2, KCNH2, KCNJ2, KCNQ1, KIF21A, KLHL40, KLHL41, KLHL9, LAMA2, LAMB2, LAMP2, LARGE1, LDB3, LDHA, LMNA, LMOD3, LPIN1, LRP4, MAG, MATR3, MEGF10, MGME1, MME, MPV17, MSTN, MTM1, MTMR14, MTO1, MUSK, MYBPC1, MYBPC3, MYF6, MYH2, MYH3, MYH6, MYH7, MYL2, MYL3, MYLK2, MYOT, MYOZ2, MYPN, NEB, NEK9, NEXN, PABPN1, PFKM, PGAM2, PGK1, PGM1, PHKA1, PHKA2, PHKB, PHKG2, PHOX2A, PHOX2B, PLEC, PLN, PNPLA2, POLG, POLG2, POMGNT1, POMGNT2, POMK, POMT1, POMT2, PRKAG2, PTRF, PYGL, PYGM, RAPSN, RBCK1, RBM20, RRM2B, RTN2, RYR1, SCN4A, SCN5A, SCO2, SEPN1, SGCA, SGCB, SGCD, SGCG, SIL1, SLC1A3, SLC22A5, SLC25A20, SLC25A3, SLC25A4, SLC2A1, SLC2A2, SLC37A4, SLC5A7, SMCHD1, SNAP25, SNTA1, SPEG, SQSTM1, STIM1, SUCLA2, SUCLG1, SYNE1, SYNE2, SYT2, TARDBP, TCAP, TIA1, TK2, TMEM43, TMEM5, TMPO, TNNC1, TNNI2, TNNI3, TNNT1, TNNT2, TNNT3, TNPO3, TNXB, TPM1, TPM2, TPM3, TRAPPC11, TRIM32, TRIP4, TRPV4, TTN, TTR, TUBB3, TYMP, UBA1, UBQLN2, VAPB, VCL, VCP, VMA21, YARS2
